# Snow Tweets: Emergency Information Dissemination in a US County During 2014 Winter Storms

**DOI:** 10.1371/currents.dis.100a212f4973b612e2c896e4cdc91a36

**Published:** 2014-12-22

**Authors:** Jess Bonnan-White, Jason Shulman, Abigail Bielecke

**Affiliations:** School of Social and Behavioral Sciences, The Richard Stockton College of New Jersey, Galloway, New Jersey, USA; Department of Physics, The Richard Stockton College of New Jersey, Galloway, New Jersey, USA; School of Social and Behavioral Sciences, The Richard Stockton College of New Jersey, Galloway, New Jersey, USA

## Abstract

Introduction: This paper describes how American federal, state, and local organizations created, sourced, and disseminated emergency information via social media in preparation for several winter storms in one county in the state of New Jersey (USA). Methods: Postings submitted to Twitter for three winter storm periods were collected from selected organizations, along with a purposeful sample of select private local users. Storm-related posts were analyzed for stylistic features (hashtags, retweet mentions, embedded URLs). Sharing and re-tweeting patterns were also mapped using NodeXL. Results: Results indicate emergency management entities were active in providing preparedness and response information during the selected winter weather events. A large number of posts, however, did not include unique Twitter features that maximize dissemination and discovery by users. Visual representations of interactions illustrate opportunities for developing stronger relationships among agencies. Discussion: Whereas previous research predominantly focuses on large-scale national or international disaster contexts, the current study instead provides needed analysis in a small-scale context. With practice during localized events like extreme weather, effective information dissemination in large events can be enhanced.

## Introduction

In recent years, scholars and practitioners of emergency management have turned their attention to the utilization of social media tools during disaster preparedness and response. Recent examples include the response to the 2010 Haiti earthquake 1 ^,^ 2 ^,^ 3, the 2010 Deepwater Horizon oil spill crisis 4, the 2010 and 2011 earthquakes in Christchurch, New Zealand 5, and the 2011 earthquake and tsunami that devastated areas of Japan 6. Much of this work has concentrated on large-scale national or international events. Smaller, more common, local disasters have not received similar scrutiny (see Dashti et al. 7). Here, we focus our attention on the use of one social media service (Twitter) for local small-scale emergency preparedness and response in one southern New Jersey county during three large snowstorms in early 2014. Our purpose is not to comprehensively review the available literature covering social media or disasters in general; instead our targeted focus is to illustrate how small-scale, localized events provide opportunity to further engage public users and evaluate gaps in information sharing prior to large crisis events.

Governmental agencies, offices of emergency management, not-for-profit organizations, and private citizens become vehicles of information distribution as they utilize services like Twitter, Facebook, Flickr, and Reddit. Previous research has focused on various aspects of *crisis informatics* 8, including mechanics of network construction, trustworthiness of information, characteristics of posts, and challenges faced by organizations in social media use during disaster response 9 ^,^ 10 ^,^ 11. For example, Smith 1demonstrated how, in the wake of the 2010 Haiti earthquake, Twitter users facilitated response activities through online information dissemination and relationship-building. Agencies and organizations, however, encounter risk of rejection by interested users, particularly potential donors, by affiliating or connecting in a deliberate way with other entities. A not-for-profit organization relying on a reputation for neutrality, for instance, may not wish to be viewed as propagating information from government sources. On the other hand, an organization might gain respect or a wider audience based on building online relationships through following and retweeting other users. Retweeting involves the rebroadcast of a message composed by another while simultaneously mentioning a particular user by their user-name or handle. In their analysis of Twitter usage during the 2010 Deepwater Horizon oil spill, Sutton et al. 4 highlighted “follow-back” activities (listing another user as a ‘favorite’) as a mechanism for opening communications and increasing believability and user trust. Muralidharan et al. 2noted differences in the emotional content of tweets during the Haiti earthquake and their potential relationship to audiences. Not-for-profit organizations seemed to employ language to reflect positive emotions (to encourage donation and additional relief efforts) whereas the media appeared to reflect negative emotions (to increase notice).

Social media services provide users almost unfettered access to information provided in “real-time” by other users able to access the service during crisis and disaster events 7 ^,^ 12. When connected to wireless or available data networks, Twitter users receive real-time streaming updates, but are limited to posts no longer than 140 characters. Several features of Twitter authorship, however, are suggested to enhance posts. These include using hashtags (terms preceded by the “#” character) to establish trending subjects, embedding URL links into the posts (as well as using URL-shortening services), and retweeting previous posts^13^. Hashtags allow users to easily search and follow a particular topic. Consistent use of hashtags would allow users to quickly access information about the storm, response, and recovery without having to search through content of multiple accounts. Hashtag usage has, in previous research, been significantly associated with variation in retweeting behavior by other users 13 ^,^ 14 ^,^ 15. Embedding links to external websites (URLs) also has been demonstrated to impact information flow and tweet survival, particularly when combined with a hashtag 13 .

Here, we investigate certain Twitter usage patterns during the immediate hours preceding, during, and following warning periods for three large 2014 snowstorms in a purposeful sampling of disaster response organizations, law enforcement units, and private citizens that service one target county. In doing so, we were interested in two specific research questions:

1) To what extent do local governmental, non-governmental, emergency management, and first-responder agencies (henceforth referred to as "official agencies") utilize unique stylistic features of Twitter (hashtags, embedded URLs, and re-tweets) to author and disseminate information?

2) To what extent do these same agencies use Twitter to share information authored by one another (by embedding URLs or mentioning user names [@+user-name]) or add their information to an existing body of tweets (using a hashtag)?

## Methods

Sampled Emergency Context

To address our research questions, Twitter usage periods covering three winter weather notices (including winter weather advisories, watches, and warnings) in March, 2014 were tracked in one county in southern New Jersey (see Table 1). Total snowfall for each storm period is noted in Table 1. We constrained our study by chronological distance (all three data collection periods are within a relatively short time span), geographic variation in response agencies and social media users (one county), and emergency context (extreme winter weather). New Jersey was considered by the authors as a state appropriate for study due to its 2013 National Health Security Preparedness Index (NHSPI) rating as among the nation’s highest in maintaining emergency public information and warning systems.^16^

**Table 1. Duration of included storm advisories, watches, and/or warnings assigned by the NWS (National Weather Service) and the expanded periods of data collection.** (*) Storm snow totals are reported in inches^17^ .

Storm	Inclusive Dates / Times of Earliest NWS Advisory, Watch, or Warning	Inclusive Twitter Collection Period	Snow Totals (*)
1	3/01/14 9:02 AM EDT (13:02 UTC)- 3/03/14 7:00 PM EDT (23:00 UTC)	2/28/14 9:02 AM EDT (13:02 UTC) - 3/04/14 1:00 AM EDT (5:00 UTC)	County Airport: 5.5 Western County: 4.5 Eastern County: 7.0
2	3/15/14 3:02 AM EDT (7:02 UTC)- 3/17/14 12:00 PM EDT (16:00 UTC)	3/14/14 3:02 AM EDT (7:02 UTC) - 3/17/14 6:00 PM EDT (22:00 UTC)	County Airport: 5.9 Western County: 4.5 Eastern County: 8.1
3	3/25/14 3:39 AM EDT (7:39 UTC)- 3/26/14 2:00 AM (6:00 UTC)	3/24/14 3:39 AM EDT (7:39 UTC) - 3/26/14 8 AM EDT (12:00 UTC)	County Airport: 3.9 Western County: 2.0/2.5 Eastern County: 4.8

A total of 17 Twitter accounts were included in the present study. Of the 570 total number of tweets made by these accounts during the three storm periods, 236 (41.4%) were determined by the authors to be storm-related. Posts were considered relevant to the current storm conditions if they 1) presented preparedness actions for the approaching storm or winter weather, 2) presented information regarding the progression of the storm (maps, snow totals, predictions), or 3) response and 4) recovery strategies (i.e., rescue deployment, school closings, road closures).

Accounts represented a variety of governmental, non-government, and private entities directly involved with emergency management information dissemination, including preparedness and response strategies. Firstly, Twitter accounts used by federal, state, county, and municipal emergency management entities working within the target county were included (see Table 2 and Appendix 1). We identified these official response organizations or agencies through a search of Twitter accounts using the following terms (individually and in combination): "[Name] County," "NJ," "New Jersey," "Emergency," "Emergency Management," and "Disaster." Additionally, names of municipalities within the target county were individually queried to locate municipal agencies. Finally, we searched for national disaster response organizations with activities serving the target county (for example, local county, municipal, or regional chapters).

The authors' own previous experience with following emergency information in the target county provided a reference for patterns of information dissemination. Although our primary research questions focused on Twitter interactions between official response organizations and government agencies, we were aware that private users and the media might also be instrumental in information dissemination. To acknowledge the potential augmentation of information dissemination by these type of users, four (4) additional accounts were included as a reference. We chose to follow the major one (1) county-specific media outlet [along with one (1) affiliated reporter who focused on emergency responses in the county]. Finally, two (2) additional accounts were identified that provided emergency information (particularly fire and police response) as it became available through public streaming scanners.

**Table 2. Description of Twitter accounts included in the present case study:** See Appendix 1 for anonymized user names.

Geographic Scope of Twitter User Activity	N
Federal	2
State	2
Regional (multi-county)	6
County	4
Municipal	3

A conscious decision was made to keep, to the furthest extent possible, names of agencies confidential in presenting the results of this case study. The intention of the authors is not to evaluate or comment on the effectiveness of individual Twitter account users. Instead, the aim was to illustrate,* at a local level* and *within a non-national disaster context, *basic interaction patterns between sources of emergency management information. Below, we discuss our results with reference to the very serious challenges of using social media tools to broadcast emergency information. Our goal was to highlight these challenges of using Twitter as a local emergency information tool and to provide comment for future refinement of its usage in a local emergency context.

Data Collection and Analysis

Data was collected using NodeXL, a network analysis and visualization software package^18^. NodeXL allows for the automatic download and import of Twitter data into a spreadsheet. Data analysis began with saving tweets generated by the accounts of interest. The final data set represents storm related tweets posted within the data collection period, including embedded URLs, included hashtags, and retweeted sources. Exploratory inferential Z-test statistical comparisons were also performed to direct future research questions. Specifically, we tested whether statistical differences in posting features between storm periods existed. For the purposes of our current study, we used a null hypothesis predicting no differences would exist between storm periods based on proportion of tweets using either hashtag, URL, or source information.

## Results

Network Mapping

Figures 1, 2, 3, 4, and 5 present visual representations of the interactions between the sampled Twitter accounts. An interaction in the figure is defined as the mentioning of an account (the “@” symbol followed by a user name) in a tweet authored by another account. For example, a common action is “retweeting,” or the broadcasting of a tweet composed by another with or without comment. Authorship is credited by the use of “RT” followed by the composing account’s handle. Interactions are illustrated in the figures by arrows originating with the author to the account that is mentioned or retweeted. Original tweets that do not mention another are represented in the figures as loops originating and terminating on the author. Consider, as an example, the lone interaction in Figure 5. The state emergency management account retweeted a message by regional office of the federal weather agency. The structure of the StateEM tweet was, “RT @FWA-Regional: [tweet text] #njwx #pawx #dewx #mdwx http:[URL]”

Figures 1 and 3 illustrate the interactions *only* between response organizations and/or county departments (non-private or media accounts) during Storms 1 and 2. Figures 2 and 4 illustrate interactions between all the sampled accounts during Storms 1 and 2. In Storm 3, there were very few tweets (N = 33) and interactions among response/government entities (only one retweeted post). There were no interactions between private accounts and response organizations. For these reasons, we only include the illustration of the total sample (Figure 5).

Nine out of 13 official accounts posted storm related tweets during the monitoring period of Storm 1. These can be observed in Figure 1 as loops starting and ending on the same account. A lone interaction was observed during this period. The StateEM account retweeted the FWA-Regional account 5 times during the monitoring period. This relatively strong interaction is represented by the dark arrow running from StateEM to FWA-Regional. However, the inclusion of the private and media accounts with the official accounts results in a more densely connected network (Figure 2). The private accounts particularly were active in retweeting or mentioning other accounts by username during the monitoring period.

Twitter activity during Storm 2 is, again, characterized by several original tweets produced by official accounts and a single interaction created by 3 retweets of FWA-Regional content by StateEM (Figure 3). Figure 4 shows that the Private 1 account retweeted or mentioned two official accounts during this time. The StateEM/FWA-Regional interaction was repeated for Storm 3, which was the sole connection between accounts (Figure 5).

**Storm 1 - Response Organization / Government Accounts:**
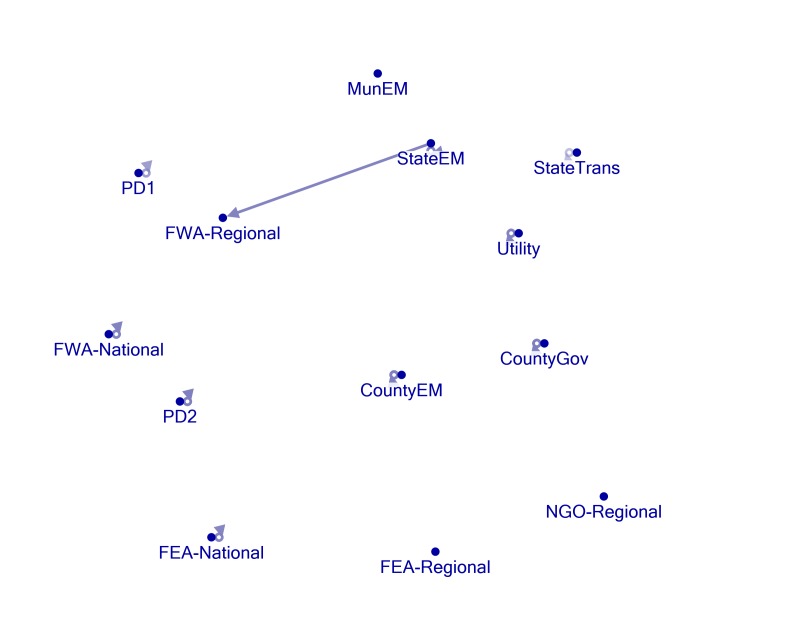
Network formed by response organization and governmental agency accounts (non-private or media) during Storm 1. Arrows originate on authors of tweets and end on accounts mentioned within them. Arrows starting and ended on the same account, i.e. loops, represent tweets that do not mention any other accounts. Arrow opacity is proportional to the number of tweets connecting the accounts and can be considered the “strength” of the connections. Note: MunEM, FEA-Regional, and NGO-Regional did not tweet about the storm, nor where they mentioned in posts by other accounts of interest. See Appendix 1 for user-name descriptions.

**Storm 1 - All Accounts:**
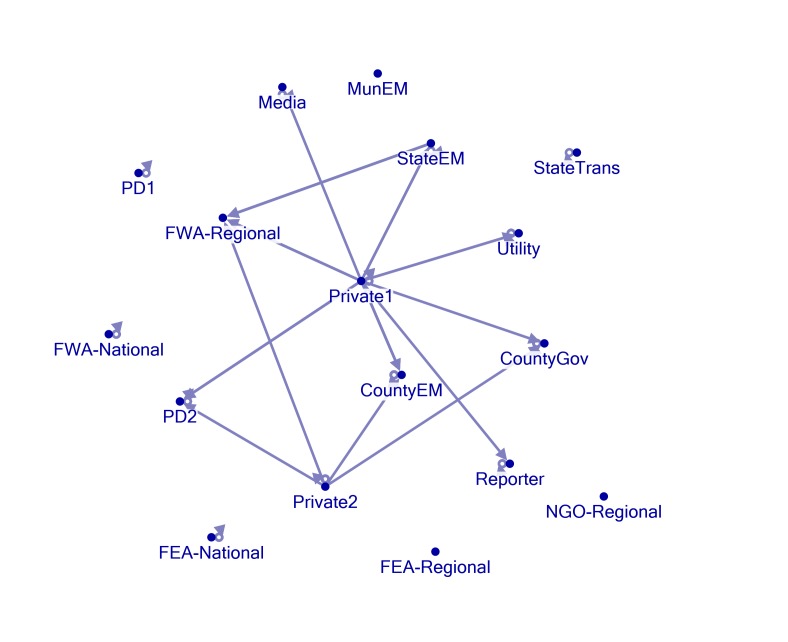
Network formed by all accounts during the monitoring period of Storm 1. See Figure 1 for figure explanations

**Storm 2 - Response Organization / Government Accounts:**
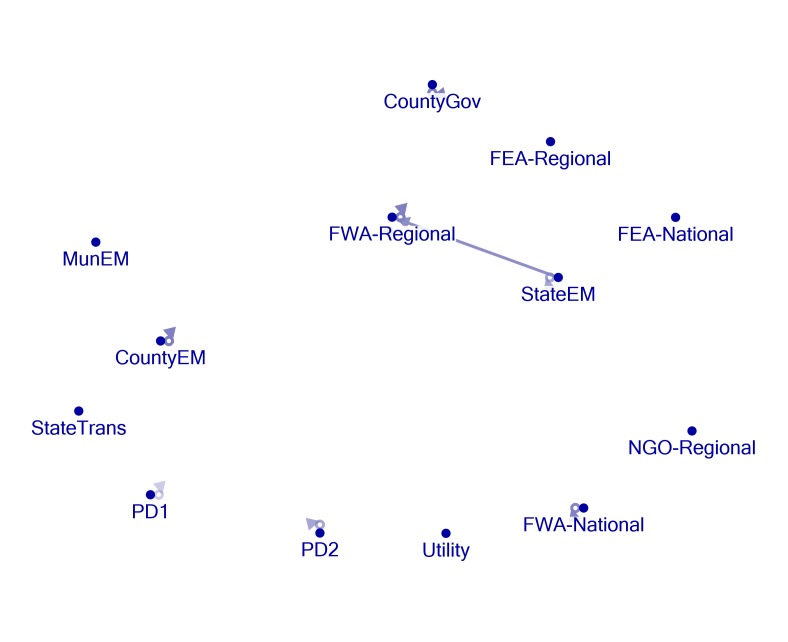
Network formed by response organization and government agency accounts (non-private or media) during Storm 2.

**Storm 2 - All Accounts:**
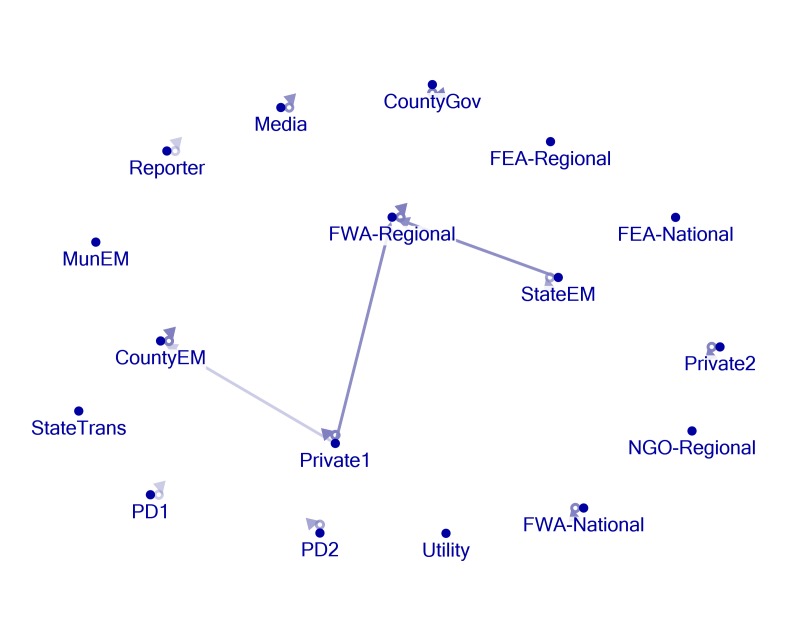
Network formed by all accounts of interest during the monitoring period of Storm 2. See Figure 1 for figure explanations.

**Storm 3 - All Accounts:**
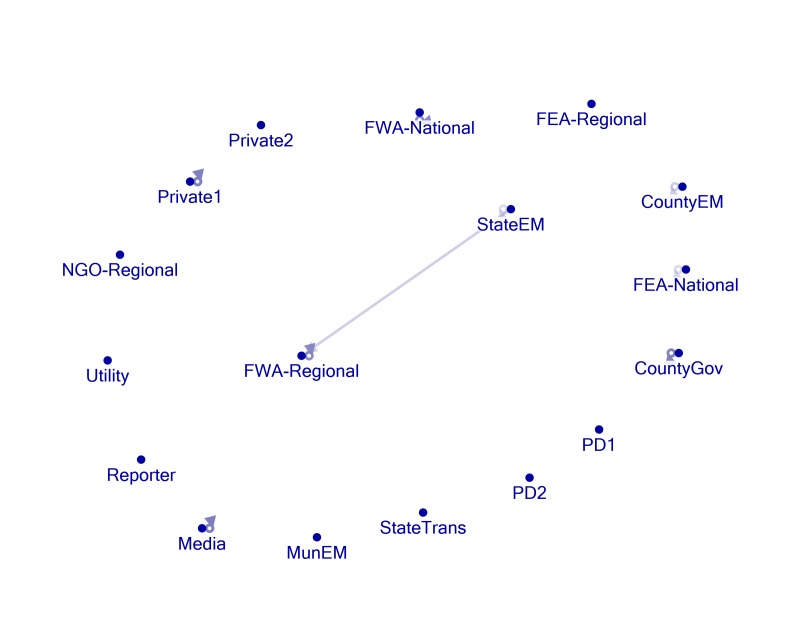
Network formed all accounts sampled during the monitoring period of Storm 3. See Figure 1 for figure explanations. Note: MunEM, FEA-Regional, and NGO-Regional did not tweet about any of the storms during the monitoring periods.

Description and Statistical Analysis of Twitter Feature Usage

Most accounts (14/17) posted at least one piece of storm-related information during at least one of the storms. The exceptions to this are the regional representative of the national disaster relief non-governmental organization, regional office of federal emergency agency, and a municipal emergency management entity. The accounts overall show relatively little interaction between one another. A minority of tweets in each storm period included retweet sources (Table 3). Notably, however, one Twitter user, the target county emergency management entity, did source information, but without the use of an “@” symbol. Such posts are categorized, therefore, as original tweets, denoted by loops on the network map figures, rather than tweets with RT mentions. The same account also regularly posted screen shots of snow accumulation prediction maps sourced from the federal weather agency. These pictures were embedded within the tweet so that followers were not required to visit an external website. A minority of tweets during each data collection period included hashtags. The most common hashtags used in the dataset consisted of abbreviations for states (for example, NJ for New Jersey) followed by wx, an acronym commonly used for referring to “extreme weather.” These hashtags included #njwx, #pawx, #mdwx, and #dewx (Table 3). Finally, a majority of the tweets posted during each storm included embedded URLs. Most frequently, the embedded URLs were used to refer followers to a larger report or explanation of current weather events. Other users also posted URL links to preparedness advice and strategies.

**Table 3. Frequency of use of common Twitter features in all storm-related tweets for each storm period.** As mentioned in the text, “wx” is an acronym that is commonly used in Twitter posts for “severe weather

Storm	Total # storm-related tweets	# Tweets with included hashtags	# Tweets with embedded URLs	# Tweets with re-tweet source
1	144	22 (15.2%)	80 (55.5%)	26 (18.1%)
2	59	15 (25.4%)	46 (83.6%)	7 (11.9%)
3	33	5 (15.2%)	28 (84.8%)	1 (3.0%)

Results of exploratory Z-statistic comparisons with a two-tailed null hypothesis *(α*=.05) are mixed. Using the proportion of tweets that included hashtags in Storm 1 (15.2%), no statistical differences were detected with Storm 2 (z=-1.7002, p=0.08914) or with Storm 3 (z=0.0182, p=0.98404). No differences were detected between the proportion of tweets using hashtags during Storm 2 (25.4%) and Storm 3 (15.2%) (z=1.1457, p=0.25014). Comparing instances of embedded URL usage, 55.5% of Storm 1 tweets included URL links. Statistical differences were found between the proportion of Storm 1 tweets and those of both Storm 2 (z=-2.988, p=0.00278) and Storm 3 (z=-3.1121, p=0.00188). No differences were found between the proportion of Storm 2 tweets (83.6%) and Storm 3 (z=-0.7981, p=0.42372) with embedded URLs. Finally, differences were found between the proportion of tweets that included a re-tweeted source in Storm 1 (18.1%) and those of Storm 3 (3%; z=2.1653, p=0.03). No differences were detected comparing Storm 1 to the Storm 2 proportion (11.9%; z=1.0855, p=0.27572) or comparing Storm 2 to Storm 3 (z=1.4423, p=0.14986). Statistical results presented here, however, should only be considered preliminary as the number of tweets (particularly during Storm 3) do not support broad interpretations.

## Discussion and Conclusion

Social media tools, including the micro-blogging site Twitter, have a potential role in different phases of the disaster management cycle. Between events (such as severe storms) preparedness information and mitigation strategies can be disseminated. During the earliest stages of response through long-term recovery, social media can also be used to identify immediate needs and point constituents to assistance. Earlier explorations of social media use during emergency periods (real-time and simulated) suggest focusing analyses on specific contexts and goals of information dissemination (see Song and Yan^19^ for example).

During escalated crisis or disaster events, responders, survivors, and external parties are inundated by the amount of information available through social media. With such an onslaught, users may transmit incomplete or incorrect information or become confused by messages from different sources^20^. News outlets may also source a story or report from information first posted onto social media, as in the case of reporters or agents following the Twitter feed of major humanitarian organizations^10^.

In the present study, there was variable hashtag and sourcing (particularly retweeting) usage. Comparing tweet features, statistical differences were found between Storm 1 and 2 in regards to the proportion that included embedded URLs. No differences were found comparing tweets with hashtags or re-tweeted sources. Readers may note that Storm 2 snowfall predictions were slightly higher in the eastern portion of the county than during Storm 1 (Table 1); statistical differences may reflect a concern on the part of Twitter users for a larger predicted snowfall during Storm 2. This is only preliminary speculation, however, and further study is required comparing (in the case of snow storms), timing of Twitter use and expected snowfall to test this hypothesis. Statistical differences were also found comparing Storm 3 to Storms 1; with the low number of tweets for the entire Storm 3 period (N=33), however, we caution any broad interpretation of these results.

Variable usage of Twitter features is not surprising, however, given Twitter accounts are not used only for crisis informatics; users must therefore balance efforts between different communication goals. Briones and colleagues^10^ noted this challenge in a discussion of American Red Cross social media use in addressing several needs, including volunteer recruitment, donor communication, and media communication. This is exacerbated by resource requirements (staff, employee hours, training, and infrastructure) to maintain timely social media presence^10^. Hughes and Palen^9^ suggest the lack of reply tweeting (which also uses the “@” and handle name) is explained by the information-sharing priority in emergency events, rather than contact with individual users. Future research can focus on discriminating between direct, reply, and retweeting behavior (all use “@” handle names) and on whether one or another type is used in certain cases to distribute information to known users who act as information hubs or distribution points.

A decline in gross posting behavior occurred during the three March 2014 winter storms monitored (Table 3). Whereas the first storm (an intense storm with high snowfall amounts; Table 1) appeared to have encouraged increased Twitter usage among the sampled users, the final storm (a less-intense event) was not characterized by a high number of storm-related Twitter posts. We suggest this may reflect a desensitization of information as users experienced each subsequent storm or as predicted intensity of the storm waned. This is an interesting pattern that deserves further study. Bruns and Burgess 5 also documented differences in Twitter use over time in relation to earthquake events in New Zealand within a 16-month period between 2010 and 2011. They suggest that moderate (but successful) usage following an earlier quake event (September 2010) primed Twitter users for even greater usage following the February 2011 earthquake. In particular, future exploration might include comparison between different natural hazard events (weather-related versus geologic events) and community attitudes towards the ability to prepare and respond to each.

Most of the official accounts actively posted storm related material during the monitoring periods, evidenced in network maps by the loops that start and end on the same account. Figures 1, 3 and 5 clearly demonstrate, however, that official accounts referred to each other very rarely using Twitter during the storm periods. In other words, while they disseminated information independently, the accounts rarely shared content produced by others via retweeting and mentioning. Only one official account consistently rejected the Twitter self-sequestration adopted by the other entities. StateEM retweeted FWA-Regional at least twice during each of the three storm periods. These are the only interactions between official accounts. Consequently, whereas official storm-related information is widely distributed throughout the various accounts, there is very little consolidation of knowledge. Interested users must closely follow each of the accounts to receive a significant portion of storm related tweets.

Although not the initial focus of the study, the private accounts played an active role in information distribution during Storms 1 and 2 (Figures 2 and 4). They were responsible for the majority of the interactions between the accounts under investigation. In fact, they served to combat the isolation practiced by the official accounts. These private accounts, therefore, were found to be amplifiers of information stemming from many of the official agencies; as such they also acted as repositories of information. Rather than following or actively searching the Twitter posts of the official accounts, a user may simply choose to follow similar accounts or browse their posts to obtain a wide array of storm related information. The behavior of these accounts highlights the importance of local amplifiers during emergency periods. However, users in search of information need to be familiar with these amplifiers if they are to quickly find information during a crisis. This difficulty can be alleviated somewhat with a search for established hashtags. Original tweets from the private accounts were not retweeted by the official entities during the monitoring period.

We recognize that social media is still geared toward a limited (albeit expanding) market. Organizations cannot ignore alternate methods of communication or risk alienating community members not currently using social media. Organizations must be continually evaluating outreach methods to particularly vulnerable populations^12^. Usage of social media may increase contact to these target communities. With the aforementioned challenges in mind, we present the following recommendations to enhance local social media presence and community interaction during periods of emergencies.

- Include encouragement to communicate between local agencies in social media training.

Retweeting messages from key local accounts might facilitate verification of data or messages by an individual Twitter user. Burnap et al.^13^ found the most important factor in the creation of a large information flow on Twitter following an event was the social portrait of the original Twitter account (for example, number of followers) (see also Morris et al.^21^). User trust in a particular disaster organization account may also increase if sources are cited. We hypothesize, both re-tweeting information from local sources and/or the citation of sources may encourage the use of Twitter an information source by community members in subsequent events. Of course, users must also be aware that unreliable information may also be distributed^6^^,^ 22 ^,^ 23 ^,^ 24 ^,^ 25 ^,^ 26; we propose, however, that with increased usage, regular users can and will learn to distinguish between sources and search for verification. We recommend that there is clear dialogue about other local users in training sessions for social media managers within organizations in order to develop an explicit, locally-based strategy on distribution of information from external sources (amplification).

- Establish and communicate hashtag usage, particularly in the hours leading up to an event if possible.

In cases where different organizations do not wish to retweet information, using a common hashtag will provide connections to individual users. For example, users in Atlantic County, New Jersey (the location of the authors) may wish to use #atlconjwx, whereas users referring to Cape May County, New Jersey could utilize #cmconjwx. Tweets that provide information about storms or events that affect multiple municipalities in these sample counties could utilize both hashtags as a way to increase distribution, and encourage retweeting^6^^,^ 27. Although more recent research has suggested hashtag usage alone may not necessarily affect the survival time of information flow about a particular event^13^, there are several variables that may impact this result. This may be due, in part, to the increase in post size (over 140 characters) with increased retweeting. Not all Twitter platforms, however, increase the size of the post with a simple retweet. We suggest these negative factors may decrease as organizational users become more familiar with Twitter tools and options (through training and exercise). Finally, Bruns and Burgess^5^ suggest the continued use of a hashtag (for example, #eqnz for ‘earthquake New Zealand’) would provide users with a pre-existing search item in the case of another event as well as a venue for continued discussion of recovery and preparedness information.

- Employ analytical tools, such as NodeXL, to periodically measure network interaction among local and relevant emergency management entities and trusted information sources.

Like other tools and strategies used in emergency management, Twitter usage should also be included in evaluation and monitoring activities. If agencies do not have staff or software resources, outreach opportunities often exist with local academic institutions with faculty who specialize in network analysis or other evaluation techniques. Although evaluation by external actors may evoke an initial sense of suspicion or worry (particularly for donor-based organizations), it is generally unnecessary, particularly if the process is started as a mutually beneficial activity. Academic partnerships often provide lower cost options that also serve to expand critical relationships and outreach potential in emergency management.

- Include multimedia objects in postings.

Burnap and colleagues^13^ found that a long survival of information flow on Twitter was positively associated with *simultaneous* embedded URL and hashtag usage. We suggest, however, whereas URL links provide access to additional information, simple pictures (such as the maps used by the target county emergency agency in this study) provide easily accessible visual data. These images may increase trust in public users (see Vis^28^). Organizational users may wish to utilize both types of posts – ones with easily identifiable, consumable information as well as posts with common hashtags and embedded URLs – to meet the multiple needs and expectations of public users. Practitioners should consider adding insight or expertise to contextualize information they share. For our purposes here, Twitter postings were recorded for a limited sample of federal, state, and local users relevant to a restricted geographic area. In the future, additional private users (including prominent meteorologists, for example) can be included in an enlarged database. Future expansion of this line of research could also include illustration of how disaster information networks grow before, during, and after an emergency event among all users of a particular hashtag. Certainly, there is justification for investigating Twitter usage in different emergency management contexts (preparedness, mitigation, response, and recovery). We did not include tweets from the sampled users that appeared to have coincided with a planned event (for example, a flood awareness or extreme weather campaign) unless the tweet directly referenced the storms. Future research might investigate how users take the opportunity to connect campaigns to current events.

Little concentration has been given to Twitter usage for common, yet relatively minor and short-term, crises like snowstorms. Other examples may include flash-flooding, traffic accidents, power outages, or public events. Most often, focus is paid in the literature to longer-term, large-scale disasters, such as earthquakes, tsunamis, wildfires, and hurricanes. Our study was intended to explore interaction between emergency response agencies during local events and illustrate opportunities to fill gaps between conceptual knowledge and practice. Local crisis events tax community resources. In both domestic and international cases, social media provides followers with information and insight into events as they unfold. With increased and enhanced communication, we hypothesize community members will increasingly engage with preparedness and response activities. Organizations can encourage greater usage by community members through utilization of Twitter features (e.g., retweeting, embedded URLs, common hashtags) that maximize both discovery by itinerant users and trust in regular users.

## Appendix 1

**Description of Twitter accounts included in the present case study:** (*) user names were removed and descriptions assigned general names to preserve a level of confidentiality.

Twitter Accounts Included in Case Study *
@FWA-Regional: Federal Weather Agency – Regional Representative
@FWA-National: Federal Weather Agency (national level)
@FEA-National: Federal Emergency Agency (national level)
@FEA-Regional: Regional Office of Federal Emergency Agency (covering target county)
@StateEM: State Emergency Agency
@CountyGov: County Government (target county)
@CountyEM: County Emergency Management Agency (target county)
@MunEM: Municipal Emergency Management Agency (in target county)
@PD1: Municipal Police Department 1 (in target county)
@PD2: Municipal Police Department 2 (in target county)
@NGO-Regional: National NGO – Regional Representative (covering target county)
@Utility: Utility Company (covering target county)
@Media: Media Outlet 1
@Reporter: Reporter (associated with Media Outlet 1)
@Private1: Private Account 1 (regularly posts county-specific emergency information)
@Private2: Private Account 2 (regularly posts county-specific emergency information)
@StateTrans: State Transportation Agency

## References

[1] Smith, B. (2010), “Socially distributing public relations: Twitter, Haiti, and interactivity in social media”, Public Relations Review, Vol. 36, pp. 329-335.

[2] Muralidharan, S., Rasmusssen, L., Patterson, D., and & Shin, J-H. (2011), “Hope for Haiti: an analysis of Facebook and Twitter usage during the earthquake relief efforts”, Public Relations Review, Vol. 37, pp. 175-177.

[3] Yates, D. and Paquette, S. (2011), “Emergency knowledge management and social media technologies: a case study of the 2010 Haitian earthquake”, International Journal of Information Management, Vol. 31, pp. 6-13.

[4] Sutton, J.N., Sprio, E.S., Johnson, B., Fitzhugh, S.M., Greczek, M., and Butts, C.T. (2012). “Connected communications: network structures of official communications in a technological disaster.” Proceedings of the 9th International ISCRAM Conference – Vancouver, Canada.

[5] Bruns, A., and Burgess, J.E. (2010). “Local and global responses to disaster: #eqnz and the Christchurch earthquake”, in Sugg, P. (Ed.) Disaster and Emergency Management Conference, Conference Proceedings – Brisbane, Australia.

[6] Acar, A., and Muraki, Y. (2011). “Twitter for crisis communication: lessons learned from Japan’s tsunami disaster”, International Journal of Web Based Communities, Vol 7 No. 3, pp. 392- 402.

[7] Dashti, S., Palen, L., Heris, M.P., Anderson, K.M., Anderson, S., and Anderson, T. J. (2014), “Supporting disaster reconnaissance with social media data: a design-oriented case study of the 2013 Colorado floods,” in Hiltz, S.R, Pfaff, M.S., Plotnick, L., and A.C. Robinson, A.C. (Eds.) Proceedings of the 11th International ISCRAM Conference – University Park, Pennsylvania, USA.

[8] Palen, L. (2008), “Online social media in crisis events”, Educause Quarterly, Vol. 31 No. 3, pp. 76-78.

[9] Hughes, A.L. and Palen, L. (2009), “Twitter adoption and use in mass convergence and emergency events,” in Landgren, J. and Jul, S. (Eds.), Proceedings of the 6th International ISCRAM Conference – Gothenburg, Sweden.

[10] Briones, R.L., Kuch, B., Liu, B.F., and Jin, Y. (2011), “Keeping up with the digital age: how the American Red Cross uses social media to build relationships”, Public Relations Review, Vol. 37, pp. 37-43.

[11] Tapia, A.H., Bajpai, K., Jansen, J., Yen, J., and Giles, L. (2011), “Seeking the trustworthy tweet: can microblogged data fit the information needs of disaster response and humanitarian relief organizations”, in Santos, M.A., Sousa, L., and Portela, E. (Eds.), Proceedings of the 8th International ISCRAM Conference – Lisbon, Portugal.

[12] Merchant, R.M., Elmer, S., and Lurie, N. (2010), “Integrating social media into emergency-preparedness efforts”, The New England Journal of Medicine, Vol. 365 No. 4, pp. 289-291. 10.1056/NEJMp1103591 21793742

[13] Burnap, P., Williams, M.L., Sloan, L., Rana, O., Housley, W., Edwards, A., Knight, V., Procter, R., and Voss, A. (2014), “Tweeting the terror: modeling the social media reaction to the Woolwich terrorist attack”, Social Network Analysis and Mining, Vol. 4 No. 1, pp. 1-14 (#206)

[14] Suh, B., Hong, L., Pirolli, P., and Chi, E.H. (2010), “Want to be retweeted? Large scale analytics on factors impacting retweet in Twitter network”, IEEE International Conference on Social Computing/ IEEE International Conference on Privacy, Security, Risk and Trust, 2010, pp. 177-184.

[15] Petrovic, S., Osborne, M., and Lavrenko, V. (2011), “RT to win! Predicting message propagation in Twitter”, Proceedings of the 5th International AAAI Conference on Weblogs and Social Media, pp. 586-589.

[16] National Health Security Preparedness Index (2014). 2013 Index Results: Index Results for New Jersey (accessed July 1, 2014).

[17] Office of the New Jersey State Climatologist 2014, Winter 2013-2014 Storm Totals (accessed August 1, 2014).

[18] Hansen, D., Shneiderman, B., and Smith, M. A. (2010), Analyzing Social Media Networks with NodeXL: Insights from a Connected World (1st ed.), Morgan Kaufmann, Burlington, MA.

[19] Song, X., and Yan, X. (2012), “Influencing factors of emergency information spreading in online social networks: a simulation approach”, Journal of Homeland Security and Emergency Management, Vol. 9 No. 1, pp. 1-14.

[20] Vieweg, S. (2010), “Microblogged contributions to the emergency arena: discovery, interpretation and implications.” CSCW – Savannah, Georgia, 2010.

[21] Morris, M.R., Counts, S., Roseway, A., Hoff, A., and Schwarz, J. (2012), “Tweeting is believing? Understanding microblog credibility perceptions”, CSCW – Seattle, Washington, 2012.

[22] Castillo, C., Mendoza, M., and Poblete, B. (2011). “Information credibility on Twitter”, Proceedings of the 2011 World Wide Web Conference – Hyderabad, India.

[23] Gupta, M., Zhao, P. , and Han, J. (2012), "Evaluating event credibility on Twitter", Proceedings of the 2012 SIAM International Conference on Data Mining - Anaheim, California

[24] Mendoza, M., Poblete, B., and Castillo, C. (2010), "Twitter under crisis: can we trust what we RT?", 1st Workshop on Social Media Analytics - Washington, DC.

[25] Mendoza, M., Poblete, B., Castillo, C. (2010), "Twitter under crisis: Can we trust what we RT?", 1st Workshop on Social Media Analytics (SOMA) - Washington, DC.

[26] Thomson, R., Ito, N., Suda, H., Lin, F., Liu, Y., Hayasaka, R., Isochi, R., and Wang, Z. (2012), "Trusting tweets: the Fukushima disaster and information source credibility on Twitter", Proceedings of the 9th International ISCRAM Conference – Vancouver, Canada.

[27] Suh, B., Hong, L., Pirolli, P., and Chi, E.H. (2010), “Want to be retweeted? Large scale analytics on factors impacting retweet in Twitter network”, IEEE International Conference on Social Computing/ IEEE International Conference on Privacy, Security, Risk and Trust, 2010, pp. 177-184.

[28] Vis, F. (2013), “Twitter as a reporting tool for breaking news”, Digital Journalism, Vol. 1 No. 1, pp. 27-47.

